# Antibiotic prophylaxis prescriptions prior to dental visits in the Veterans’ Health Administration (VHA), 2015–2019

**DOI:** 10.1017/ice.2021.521

**Published:** 2022-11

**Authors:** Katie J. Suda, Margaret A. Fitzpatrick, Gretchen Gibson, M. Marianne Jurasic, Linda Poggensee, Kelly Echevarria, Colin C. Hubbard, Jessina C. McGregor, Charlesnika T. Evans

**Affiliations:** 1 Center for Health Equity Research and Promotion, Department of Veterans’ Affairs (VA), VA Pittsburgh Healthcare System, Pittsburgh, Pennsylvania; 2 Division of General Internal Medicine, Department of Medicine, University of Pittsburgh School of Medicine, Pittsburgh, Pennsylvania; 3 Center of Innovation for Complex Chronic Healthcare, Edward Hines, Jr VA Hospital, Hines, Illinois; 4 Division of Infectious Diseases, Department of Medicine, Loyola University Chicago Stritch School of Medicine, Maywood, Illinois; 5 Veterans’ Health Administration Office of Dentistry, Washington, DC; 6 Boston University Henry M. Goldman School of Dental Medicine, Boston, Massachusetts; 7 VA Center for Healthcare Organization and Implementation Research, Edith Nourse Rogers Memorial Veterans Hospital, Bedford, Massachusetts; 8 Antimicrobial Stewardship Task Force, Pharmacy Benefits Management Program, Department of Veterans Affairs, Washington, DC; 9 Division of Hospital Medicine, Department of Medicine, University of California San Francisco, San Francisco, California; 10 Oregon State University College of Pharmacy, Portland, Oregon; 11 Department of Preventive Medicine, Center for Health Services and Outcomes Research, Institute for Public Health and Medicine, Feinberg School of Medicine, Northwestern University, Chicago, Illinois

## Abstract

**Objective:**

To determine prophylaxis appropriateness by Veterans’ Affairs (VA) dentists.

**Design:**

A cross-sectional study of dental visits, 2015–2019.

**Methods:**

Antibiotics within 7 days before a visit in the absence of an oral infection were included. Appropriate antibiotic prophylaxis was defined as visits with gingival manipulation and further delineated into narrow and broad definitions based on comorbidities. The primary analysis applied a narrow definition of appropriate prophylaxis: cardiac conditions at the highest risk of an adverse outcome from endocarditis. The secondary analysis included a broader definition: cardiac or immunocompromising condition or tooth extractions and/or implants. Multivariable log-linear Poisson generalized estimating equation regression was used to assess the association between covariates and unnecessary prophylaxis prescriptions.

**Results:**

In total, 358,078 visits were associated with 369,102 antibiotics. The median prescription duration was 7 days (IQR, 7–10); only 6.5% were prescribed for 1 day. With the narrow definition, 15% of prophylaxis prescriptions were appropriate, which increased to 72% with the broader definition. Prophylaxis inconsistent with guidelines increased over time. For the narrow definition, Black (vs White) race, Latine (vs non-Latine) ethnicity, and visits located in the West census region were associated with unnecessary prophylaxis. Variables associated with a lower risk were older age, prosthetic joints, immunocompromising condition, and rural location.

**Conclusions:**

Of every 6 antibiotic prophylaxis prescriptions, 5 were inconsistent with guidelines. Improving prophylaxis appropriateness and shortening duration may have substantial implications for stewardship. Guidelines should state whether antibiotic prophylaxis is indicated for extractions, implants, and immunocompromised patients.

Combating antimicrobial resistance through antibiotic stewardship is a priority.^1^ One component of stewardship is monitoring appropriate prescribing. Nationally, antibiotic prescribing rates are decreasing, but they are increasing for advanced practice providers and dentists.^2^ Dentists prescribe 10% of antibiotics, and they lead clindamycin prescribing.^3,4^ Dental antibiotics have been associated with adverse events including hospitalizations, emergency department visits, and *C. difficile*.^5,6^

Antibiotic prophylaxis guidelines for dental procedures are lacking. Guidelines do recommend prophylaxis prior to invasive procedures in those at highest risk of an adverse outcome from endocarditis.^7^ Other guidelines discourage prophylaxis in patients with prosthetic joints.^8^ Regarding specific procedures, Cochrane meta-analyses have demonstrated that a single antibiotic dose prior to a tooth extraction or dental implant placement may decrease the risk of postprocedure infection and implant failure.^9,10^ Whether these benefits outweigh the risk of adverse events remains unclear, especially with longer treatment courses.^6,9,10^ Within dentistry, it is common for prophylaxis to be prescribed in patients with medically complex conditions (eg, immunosuppression) prior to invasive dental procedures,^11^ despite a lack of data on the benefit of such practice.

Determining the appropriateness of antibiotics prescribed by dentists has been challenging because dentists code using comprehensive dental terminology (CDT) codes and rarely use diagnostic codes used in medical fields [eg, *International Classification of Diseases, Ninth Revision, Clinical Modification* (ICD-9-CM) or *Tenth Revision* (ICD-10-CM)], where research on antibiotic appropriateness has focused.^12^ Thus, it is difficult to associate a prescription with a diagnosis. Dentists in the Veterans’ Health Administration (VHA) use both CDT and ICD9/10 codes. Thus, this setting provides an opportunity to accurately assess prophylaxis prescribing by removing antibiotics associated with an oral infection. Determining antibiotic appropriateness to inform stewardship efforts is important because most dental prescriptions are for antibiotics.^4^ Thus, we sought to determine the appropriateness of antibiotic prophylaxis prescribed by VHA dentists and to identify factors associated with unnecessary prophylaxis.

## Methods

### Study design and setting

We conducted a cross-sectional study of dental visits from 2015–2019 using the national VHA Corporate Data Warehouse (CDW) database. From the CDW, we collected patient demographics, diagnoses (ICD-9/ICD-10-CM), prescriptions dispensed from VHA pharmacies, and dental visit characteristics (ICD-9/ICD-10-CM/CDT). For all analyses, ICD-9 codes assigned before October 1, 2015, were converted to ICD-10-CM codes according to Centers for Disease Control and Prevention (CDC) guidance.^13^ Codes documented within 1 year prior to the dental visit were used to construct a Charlson comorbidity index, Charlson comorbidity categories, and an Elixhauser comorbidity index. Race and ethnicity were self-defined by veterans; those selecting multiple races were categorized as multiracial.^14^ The dates of analysis were January 2020–April 2021. The Hines VA Investigational Review Board granted this study expedited approval.

### Study population

Prescriptions were identified in the CDW pharmacy domains; dental diagnoses and procedures were identified in the dental domain; and comorbidities were identified in the outpatient and inpatient domains. Prescriptions and dental diagnoses and/or procedures were collected from 2015 to 2019. Comorbidities were identified in the records from 1992 until prior to the antibiotic prescription associated with the dental visit. Only antibiotics prescribed by a dentist were included. Eligible patients were veterans with a dental visit and a systemic antibiotic dispensed within 7 days before the visit. These antibiotics were defined as being prescribed for preprocedural infection prophylaxis. Using ICD-9/ICD-10-CM codes for oral infections, veterans with an oral infection diagnosis associated with the antibiotic were excluded (Supplementary Table 1 online). These criteria are consistent with prior studies and are based upon manual review of dental records and expert guidance.^15^ However, a history of pulpitis, periodontitis, and/or acute apical abscess were defined in accord with previous work.^16^ Dental clinics were categorized within Census Bureau regions, with clinics in Puerto Rico categorized as “US territories.” VHA facilities in which dental clinics were located were further categorized by complexity: levels 1a–c facilities are high complexity and levels 2–3 are low complexity. The complexity of a facility is based on patient characteristics, clinical programs, and teaching programs.

**Table 1.** Unadjusted Associations Between Demographic and Medical Characteristics and Unnecessary Antibiotic Prophylaxis Prescriptions According to the Narrow Criteria^a,^^b^

Variable	Value	Total, No. (%)	Appropriate (Narrow Criteria) (N = 53,442), No. (%)	Unnecessary (N = 304,636), No. (%)	Prevalence Rate Ratio (95% CI)	*P* Value
Year	2015	71,935 (20.1)	11,479 (16)	60,456 (84)	Reference	
	2016	74,297 (20.7)	11,502 (15.5)	62,795 (84.5)	1.0057 (1.0012–1.0101)	.0124
	2017	72,874 (20.4)	10,914 (15)	61,960 (85)	1.0117 (1.0072–1.0161)	<.0001
	2018	70,651 (19.7)	10,017 (14.2)	60,634 (85.8)	1.0212 (1.0167–1.0256)	<.0001
	2019	68,321 (19.1)	9,530 (13.9)	58,791 (86.1)	1.0239 (1.0194–1.0284)	<.0001
Gender	Male	325,763 (91)	49,722 (15.3)	276,041 (84.7)	Reference	
	Female	32,315 (9)	3,720 (11.5)	28,595 (88.5)	1.0443 (1.0399–1.0487)	<.0001
Race	White	241,747 (67.5)	38,810 (16.1)	202,937 (83.9)	Reference	
	Black	89,475 (25)	11,303 (12.6)	78,172 (87.4)	1.0408 (1.0376–1.0439)	<.0001
	Native American/Alaskan	2,992 (0.8)	423 (14.1)	2,569 (85.9)	1.0228 (1.008–1.0379)	.0043
	Native Hawaiian/Pacific Islander	3,759 (1)	507 (13.5)	3,252 (86.5)	1.0306 (1.0175–1.0438)	<.0001
	Asian	3,978 (1.1)	422 (10.6)	3,556 (89.4)	1.0649 (1.0534–1.0765)	<.0001
	Multiracial	4,064 (1.1)	540 (13.3)	3,524 (86.7)	1.033 (1.0205–1.0456)	<.0001
	Missing	12,063 (3.4)	1,437 (11.9)	10,626 (88.1)		
Ethnicity	Non-Latine	32,1821 (89.9)	48,795 (15.2)	273,026 (84.8)	Reference	
	Latine	29,268 (8.2)	3,688 (12.6)	25,580 (87.4)	1.0302 (1.0255–1.0349)	<.0001
	Missing	6,989 (2)	959 (13.7)	6,030 (86.3)		
Age	18–44 y	38,447 (10.7)	1,824 (4.7)	36,623 (95.3)	Reference	
	45–64 y	128,429 (35.9)	14,676 (11.4)	113,753 (88.6)	0.9298 (0.9271–0.9326)	<.0001
	≥65 y	191,202 (53.4)	36,942 (19.3)	154,260 (80.7)	0.847 (0.8443–0.8496)	<.0001
Rural		40,025 (11.2)	6,635 (16.6)	33,390 (83.4)	0.9778 (0.9733–0.9823)	<.0001
	Missing	1,1931 (3.3)	1,866 (15.6)	10,065 (84.4)		
Complexity	1a/1b/1c	308,582 (86.2)	45,357 (14.7)	263,225 (85.3)	Reference	
	2	22,933 (6.4)	3,753 (16.4)	19180 (83.6)	0.9805 (0.9747–0.9863)	<.0001
	3	26,563 (7.4)	4,332 (16.3)	22,231 (83.7)	0.9811 (0.9757–0.9865)	<.0001
Region	Northeast	51817 (14.5)	7848 (15.1)	43969 (84.9)	Reference	
	Midwest	67,002 (18.7)	11,950 (17.8)	55,052 (82.2)	0.9683 (0.9634–0.9732)	<.0001
	South	160,298 (44.8)	22,454 (14)	137,844 (86)	1.0134 (1.0092–1.0176)	<.0001
	West	72,967 (20.4)	10,301 (14.1)	62,666 (85.9)	1.0121 (1.0074–1.0169)	<.0001
	US territories	5,994 (1.7)	889 (14.8)	5,105 (85.2)	1.0037 (0.9925–1.015)	.5297
Smoking	Never smoked	68,945 (19.3)	10,677 (15.5)	58,268 (84.5)	Reference	
	Current smoker	10,4679 (29.2)	15,160 (14.5)	89,519 (85.5)	1.0119 (1.0078–1.016)	<.0001
	Past smoker	59,826 (16.7)	10,762 (18)	49,064 (82)	0.9704 (0.9656–0.9752)	<.0001
	Missing	124,628 (34.8)	16,843 (13.5)	107,785 (86.5)		
Patient characteristic	Friday visit	61,730 (17.2)	8,887 (14.4)	52,843 (85.6)	1.0075 (1.0039–1.0111)	<.0001
	Cardiac condition	60,428 (16.9)	53,442 (88.4)	6,986 (11.6)	0.1156 (0.1131–0.1182)	<.0001
	Prosthetic condition	92,522 (25.8)	17,143 (18.5)	75,379 (81.5)	0.9437 (0.9405–0.9469)	<.0001
	Immunocompromised	242,093 (67.6)	42,402 (17.5)	199,691 (82.5)	0.9116 (0.9092–0.914)	<.0001
	History of pulpitis	200,744 (56.1)	31,437 (15.7)	169,307 (84.3)	0.9805 (0.9779–0.9832)	<.0001
	History of periodontitis	28,117 (7.9)	4,365 (15.5)	23,752 (84.5)	0.9924 (0.9872–0.9975)	.0034
	History of acute apical abscess	11,132 (3.1)	1,508 (13.5)	9,624 (86.5)	1.0167 (1.0091–1.0244)	<.0001
Elixhauser index	0	121,110 (33.8)	9,436 (7.8)	111,674 (92.2)	Reference	
	1	97,805 (27.3)	12,906 (13.2)	84,899 (86.8)	0.9414 (0.9386–0.9442)	<.0001
	2	139,163 (38.9)	31,100 (22.3)	108,063 (77.7)	0.8421 (0.8394–0.8449)	<.0001
Extraction	No extraction	253,724 (70.9)	36,794 (14.5)	216,930 (85.5)	Reference	
	Simple extraction	51,393 (14.4)	8,327 (16.2)	43,066 (83.8)	0.9801 (0.9761–0.9842)	<.0001
	Surgical extraction	52,961 (14.8)	8,321 (15.7)	44,640 (84.3)	0.9858 (0.9819–0.9898)	<.0001
Invasive dental visit	Routine	125,091 (34.9)	19,497 (15.6)	105,594 (84.4)	Reference	
	Mildly invasive	40,824 (11.4)	5,657 (13.9)	35,167 (86.1)	1.0205 (1.0158–1.0252)	<.0001
	Invasive	192,163 (53.7)	28,288 (14.7)	163,875 (85.3)	1.0103 (1.0072–1.0133)	<.0001
Amoxicillin/ Clavulanic acid		18,269 (5.1)	2,178 (11.9)	16,091 (88.1)	1.0373 (1.0316–1.043)	<.0001
Clindamycin		57,999 (16.2)	9,581 (16.5)	48,418 (83.5)	0.9777 (0.9739–0.9815)	<.0001
Azithromycin		3,937 (1.1)	525 (13.3)	3,412 (86.7)	1.0189 (1.0064–1.0315)	.0046
Penicillin		23,197 (6.5)	2,840 (12.2)	20,357 (87.8)	1.0338 (1.0286–1.039)	<.0001
Doxycycline		2,494 (0.7)	313 (12.6)	2,181 (87.4)	1.0281 (1.0129–1.0436)	.0007
Metronidazole		2,385 (0.7)	269 (11.3)	2,116 (88.7)	1.0432 (1.0283–1.0583)	<.0001

a Cardiac condition + gingival manipulation.

b The following variables were nonsignificant and removed from the table: amoxicillin, cephalexin, fluoroquinolone, and other antibiotic. The complete tables with the Charlson scores and disease categories are included in Supplementary Table 3 (online). Ceftriaxone was not prescribed during the study period. Where not indicated, the reference group is in absence of that condition. For example, the reference group for Friday visit are visits occurring on all other days and the reference group for clindamycin are non–clindamycin antibiotics.

Similar to prior work,^15^ we combined dental visits into a single observation (or an episode of care) where all codes from each visit were represented as a single episode of care because dental care is commonly delivered sequentially over multiple visits (Supplementary Fig. 1 online). For example, a tooth extraction is diagnosed at one visit, but the extraction procedure is performed at a second visit. Thus, we combined all dental visits occurring within 14 days after the index visit into a single episode of care. A dental visit occurring within 14 days of a prior dental visit but with an intervening antibiotic started a new episode of care. Clustering visits merged 141,131 subsequent visits into an episode of care with an earlier visit (26.2% had 1 visit and 4.1% had ≥2 visits). Broadening the episode-of-care definition to 30 days linked visits that were unlikely to be related clinically and captured few additional visits. Episodes of care are reported as visits herein, but they are, in fact, visits collapsed into episodes of care as described.

**Fig. 1.**
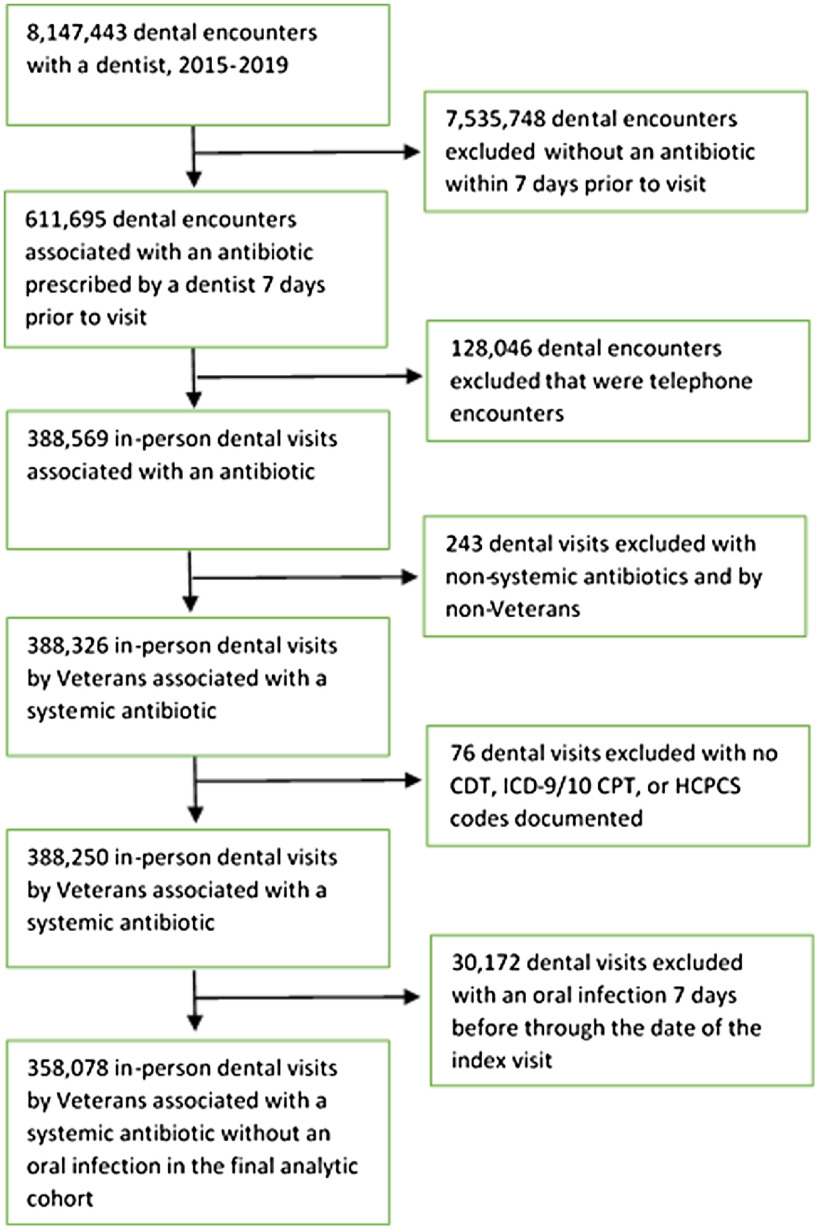
Flowchart of study cohort of veteran dental visits associated with antibiotic prophylaxis.

### Study definitions

In this study, antibiotic prophylaxis was only considered appropriate if the prescription was associated with a dental visit that involved manipulation of gingival tissue or the periapical region of teeth or perforation of the oral mucosa, referred to herein as gingival manipulation.^7^ Gingival manipulation was determined for each CDT, CPT, and HCPCS associated with dental visits.^15^ Appropriate antibiotic prophylaxis was then further delineated into narrow and broad definitions by comorbidities and dental procedure. The narrow definition, the primary analysis, defined antibiotic prophylaxis as appropriate if the patient had a cardiac condition at the highest risk of an adverse outcome from infective endocarditis according to guidelines.^7^ Due to the difficulty in distinguishing valvulopathy and specific types of congenital heart disease, we included all cardiac transplant (with and without valvulopathy) and all congenital heart disease patients. The broad definition defined antibiotic prophylaxis as appropriate if the patient had a cardiac condition, or an immunocompromising condition (Supplementary Table 2 online), or if the procedure was a surgical tooth extraction or dental implant placement.^11,17–19^ Extraction CDTs were further delineated into simple and surgical extractions.^20^ Surgical extractions included the incision of gingiva and bone removal and are considered more invasive. Using a hierarchical approach, each visit was defined as invasive, mildly invasive, or routine. Invasive CDTs were considered the highest level of invasiveness and included visits with codes for oral surgery, periodontics, endodontics, or dental implants. Visits not in the invasive category with restorative, prosthodontics, or maxillofacial prosthetics CDTs were categorized as mildly invasive. Visits without these categories coded and with diagnostic, preventive, adjunct, orthodontic CDTs were defined as routine. Appropriate antibiotic selection was defined consistent with guidelines as amoxicillin, ampicillin, azithromycin, ceftriaxone, cephalexin, clarithromycin, and clindamycin.^7^

**Table 2.** Frequency of Antibiotic Prescription Duration Stratified by Day Equal to 1 Day Prescription Duration or ≥2 Day Prescription Duration

Year	1-Day Prescription Duration, No. (%)	≥2-Day Prescription Duration, No. (%)	Total, No. (%)
2015	4,547 (6.3)	67,388 (93.7)	71,935 (20.1)
2016	4,727 (6.4)	69,570 (93.6)	74,297 (20.8)
2017	4,668 (6.4)	68,206 (93.6)	72,874 (20.4)
2018	4,600 (6.5)	66,051 (93.5)	70,651 (19.7)
2019	4,632 (6.8)	63,689 (93.2)	68,321 (19.1)
Total	23,174 (6.5)	334,904 (93.5)	358,078 (100)

In the absence of other indications meeting the narrow or broad definition, antibiotic prophylaxis in patients with prosthetic joints were considered unnecessary.^8,15^ Guidelines published in 2003 recommended prophylaxis 2 years after joint placement (the highest-risk period for infection) and in patients with prosthetic joints and an additional high-risk comorbidity (eg, cancer).^17^ Given that guidance changed shortly before the study period, sensitivity analyses were conducted, varying the definition of an indication for joint-related prophylaxis: (1) defining a 2-year window after joint placement and (2) using prior guidelines.^17^ Other sensitivity analyses estimated appropriateness by varying selection criteria (1) removing the gingival manipulation criteria and (2) stratifying patients with and without prosthetic joints. The gingival manipulation criteria were removed due to a lack of validation using the assembled codes and/or to account for the possibility of missing codes.

### Statistical analysis

Descriptive characteristics of visits with and without unnecessary antibiotic prophylaxis were compared using independent *t* tests for continuous variables and χ^2^ tests for categorical variables. Missing data were included in the analysis and are labeled as missing. The association of covariates with a count of visits with unnecessary prophylaxis (dependent variable) was modeled using multivariable log-linear Poisson generalized estimating equation regression, which accounted for clustering by patient. The models calculated adjusted prevalence rate ratios (PR) and 95% confidence intervals (CIs) with robust standard errors for association between patient-level and visit-level characteristics and appropriate prophylaxis. Variables significant in unadjusted analyses, defined as a *P* value <.10, were included in the multivariable models. To reach the most parsimonious model, a backward selection procedure removed nonsignificant variables to identify final variables significantly associated with unnecessary prophylaxis prescriptions. Stata software (StataCorp, College Station, TX) and SAS version 9.4 software (SAS Institute, Cary, NC) were used for these analyses. A priori hypothesis tests were performed with a 2-sided α = .05.

## Results

During the study period, 388,250 visits (229,439 patients) were associated with 400,626 antibiotics prescriptions (Fig. 1). In 8% of the visits associated with ≥1 antibiotic, antibiotics were prescribed for an oral infection and the visit was excluded. The remaining 92% met study definitions for a total of 369,102 prophylaxis prescriptions for 358,078 visits (mean, 1.03 antibiotics per visit). In our cohort, prophylaxis was prescribed to 218,231 patients (91% male; mean age, 61.2 years) (Table 1). Amoxicillin comprised most antibiotic prophylaxis (74.8%), followed by clindamycin (16.2%). Antibiotic prophylaxis were prescribed for a median of 7 days (IQR, 7–10; mean, 8.4±8.1 days); 28.2% were prescribed for ≥10 days, and only 6.5% were prescribed for 1 day (Table 2).

### Unadjusted analysis

Overall, 90% of dental visits associated with antibiotic prophylaxis prescriptions were categorized as gingival manipulation. With the narrow definition, 85% of prophylaxis prescriptions were inconsistent with guidelines. In the unadjusted analysis, significant associations were identified between guideline-concordant prophylaxis for patient characteristics, visit timing, procedure invasiveness, and location (Table 1). More than 91% of prescriptions were with an appropriately selected antibiotic according to the guidelines.^7^ Adding dental implants, surgical tooth extractions, and immunocompromising conditions to the cardiac criteria, 28% of antibiotics were defined as unnecessary per the broad definition. Using this broad definition of appropriateness, unadjusted predictors included patient characteristics, visit timing, procedure invasiveness, and location (Table 3).

**Table 3.** Unadjusted Associations Between Demographic and Medical Characteristics and Unnecessary Antibiotic Prophylaxis According to the Broad Criteria^a,b^

Variable	Value	Total, No. (%)	Appropriate (Broad Criteria) (N = 256,591), No. (%)	Inappropriate (N = 101,487), No. (%)	Prevalence Rate Ratio (95% CI)	*P* Value
Year	2015	71,935 (20.1)	53,752 (74.7)	18,183 (25.3)	Reference	
	2016	74,297 (20.7)	54,067 (72.8)	20,230 (27.2)	1.0772 (1.0588–1.0959)	<.0001
	2017	72,874 (20.4)	51,041 (70)	21,833 (30)	1.1853 (1.1656–1.2053)	<.0001
	2018	70,651 (19.7)	49,577 (70.2)	21,074 (29.8)	1.1801 (1.1603–1.2002)	<.0001
	2019	68,321 (19.1)	48,154 (70.5)	20,167 (29.5)	1.1678 (1.148–1.1879)	<.0001
Gender	Male	325,763 (91)	233,164 (71.6)	92,599 (28.4)	Reference	
	Female	32,315 (9)	23,427 (72.5)	8,888 (27.5)	0.9676 (0.9498–0.9857)	.0005
Race	White	241,747 (67.5)	173,994 (72)	67,753 (28)	Reference	
	Black	89,475 (25)	64,039 (71.6)	25,436 (28.4)	1.0143 (1.002–1.0268)	.0226
	Native American/Alaskan	2,992 (0.8)	2,125 (71)	867 (29)	1.0339 (0.9772–1.094)	.2515
	Native Hawaiian/Pacific Islander	3,759 (1)	2,687 (71.5)	1,072 (28.5)	1.0175 (0.9669–1.0708)	.5101
	Asian	3,978 (1.1)	2,762 (69.4)	1,216 (30.6)	1.0907 (1.0403–1.1435)	.0005
	Multiracial	4,064 (1.1)	2,983 (73.4)	1,081 (26.6)	0.9491 (0.9015–0.9992)	.0446
	Missing	12,063 (3.4)	8,001 (66.3)	4,062 (33.7)		
Ethnicity	Non-Latine	321,821 (89.9)	230,000 (71.5)	91,821 (28.5)	Reference	
	Latine	29,268 (8.2)	21,837 (74.6)	7,431 (25.4)	0.8899 (0.8719–0.9082)	<.0001
	Missing	6,989 (2)	4,754 (68)	2,235 (32)		
Age	18–44 y	38,447 (10.7)	25,219 (65.6)	13,228 (34.4)	Reference	
	45–64 y	128,429 (35.9)	92,231 (71.8)	36,198 (28.2)	0.8192 (0.8059–0.8327)	<.0001
	≥65 y	191,202 (53.4)	139,141 (72.8)	52,061 (27.2)	0.7914 (0.7791–0.8038)	<.0001
Complexity	1a/1b/1c	308,582 (86.2)	222,542 (72.1)	86,040 (27.9)	Reference	
	2	22,933 (6.4)	16,115 (70.3)	6,818 (29.7)	1.0663 (1.0444–1.0886)	<.0001
	3	26,563 (7.4)	17,934 (67.5)	8,629 (32.5)	1.1651 (1.144–1.1865)	<.0001
Region	Northeast	51,817 (14.5)	36,845 (71.1)	14,972 (28.9)	Reference	
	Midwest	67,002 (18.7)	48,882 (73)	18,120 (27)	0.936 (0.9189–0.9533)	<.0001
	South	160,298 (44.8)	114,718 (71.6)	45,580 (28.4)	0.9841 (0.9689–0.9996)	.0446
	West	72,967 (20.4)	51,386 (70.4)	21,581 (29.6)	1.0236 (1.0058–1.0417)	.0091
	US Territories	5,994 (1.7)	4,760 (79.4)	1,234 (20.6)	0.7125 (0.6767–0.7502)	<.0001
Smoking	Never smoked	68,945 (19.3)	49,964 (72.5)	18,981 (27.5)	Reference	
	Current smoker	104,679 (29.2)	79,293 (75.7)	25,386 (24.3)	0.8809 (0.8668–0.8952)	<.0001
	Past smoker	59,826 (16.7)	44,724 (74.8)	15,102 (25.2)	0.9169 (0.9002–0.9339)	<.0001
	Missing	124,628 (34.8)	82,610 (66.3)	42,018 (33.7)		
Patient characteristic	Friday visit	61,730 (17.2)	43,390 (70.3)	18,340 (29.7)	1.0589 (1.0448–1.0732)	<.0001
	Cardiac condition	60,428 (16.9)	53,442 (88.4)	6,986 (11.6)	0.3641 (0.356–0.3725)	<.0001
	Prosthetic condition	92,522 (25.8)	66,818 (72.2)	25,704 (27.8)	0.9735 (0.9619–0.9853)	<.0001
	Immunocompromised	242,093 (67.6)	217,912 (90)	24,181 (10)	0.1499 (0.148–0.1518)	<.0001
	History of pulpitis	200,744 (56.1)	146,237 (72.8)	54,507 (27.2)	0.9093 (0.8999–0.9189)	<.0001
	History of periodontitis	28,117 (7.9)	20,615 (73.3)	7,502 (26.7)	0.9367 (0.9181–0.9558)	<.0001
Elixhauser index	0	121,110 (33.8)	69,682 (57.5)	51,428 (42.5)	Reference	
	1	97,805 (27.3)	71,398 (73)	26,407 (27)	0.6358 (0.6281–0.6436)	<.0001
	2	139,163 (38.9)	115,511 (83)	23,652 (17)	0.4002 (0.3949–0.4056)	<.0001
Charlson score	0	267,437 (74.7)	183,725 (68.7)	83,712 (31.3)	Reference	
	1	25,775 (7.2)	19,903 (77.2)	5,872 (22.8)	0.7278 (0.7111–0.7449)	<.0001
	2 or more	64,866 (18.1)	52,963 (81.6)	11,903 (18.4)	0.5862 (0.5763–0.5964)	<.0001
Extraction	No extraction	253,724 (70.9)	166,316 (65.5)	87,408 (34.5)	Reference	
	Simple extraction	51,393 (14.4)	37,314 (72.6)	14,079 (27.4)	0.7952 (0.7833–0.8073)	<.0001
	Surgical extraction	52,961 (14.8)	52,961 (100)	0 (0)	…	<.0001
Invasive visit	Routine	125,091 (34.9)	78,108 (62.4)	46,983 (37.6)	Reference	
	Mildly invasive	40,824 (11.4)	19,027 (46.6)	21,797 (53.4)	1.4216 (1.4053–1.4381)	<.0001
	Invasive	192,163 (53.7)	159,456 (83)	32,707 (17)	0.4532 (0.4477–0.4587)	<.0001
Amoxicillin		267,826 (74.8)	192414 (71.8)	75,412 (28.2)	0.9746 (0.9631–0.9862)	<.0001
Amoxicillin/ clavulanic acid		18,269 (5.1)	13,222 (72.4)	5,047 (27.6)	0.9734 (0.9503–0.9971)	.0279
Clindamycin		57,999 (16.2)	42,024 (72.5)	15,975 (27.5)	0.9666 (0.9528–0.9805)	<.0001
Cephalexin		2,888 (0.8)	1,901 (65.8)	987 (34.2)	1.2079 (1.1479–1.2709)	<.0001
Penicillin		23,197 (6.5)	16,118 (69.5)	7,079 (30.5)	1.0825 (1.0609–1.1045)	<.0001
Doxycycline		2,494 (0.7)	1,651 (66.2)	843 (33.8)	1.1942 (1.1301–1.262)	<.0001
Metronidazole		2,385 (0.7)	1,584 (66.4)	801 (33.6)	1.1865 (1.1211–1.2556)	<.0001
Other antibiotic		529 (0.1)	355 (67.1)	174 (32.9)	1.1608 (1.0277–1.3112)	.0232

a Cardiac condition or surgical extraction/tooth implant or immunocompromised + gingival manipulation).

b The following variables were nonsignificant and were removed from the table: rural, history of acute apical abscess, azithromycin, and fluoroquinolone. The complete tables with the Charlson scores and disease categories are included in Supplementary Table 4 (online).

### Adjusted analysis

**Fig. 2.**
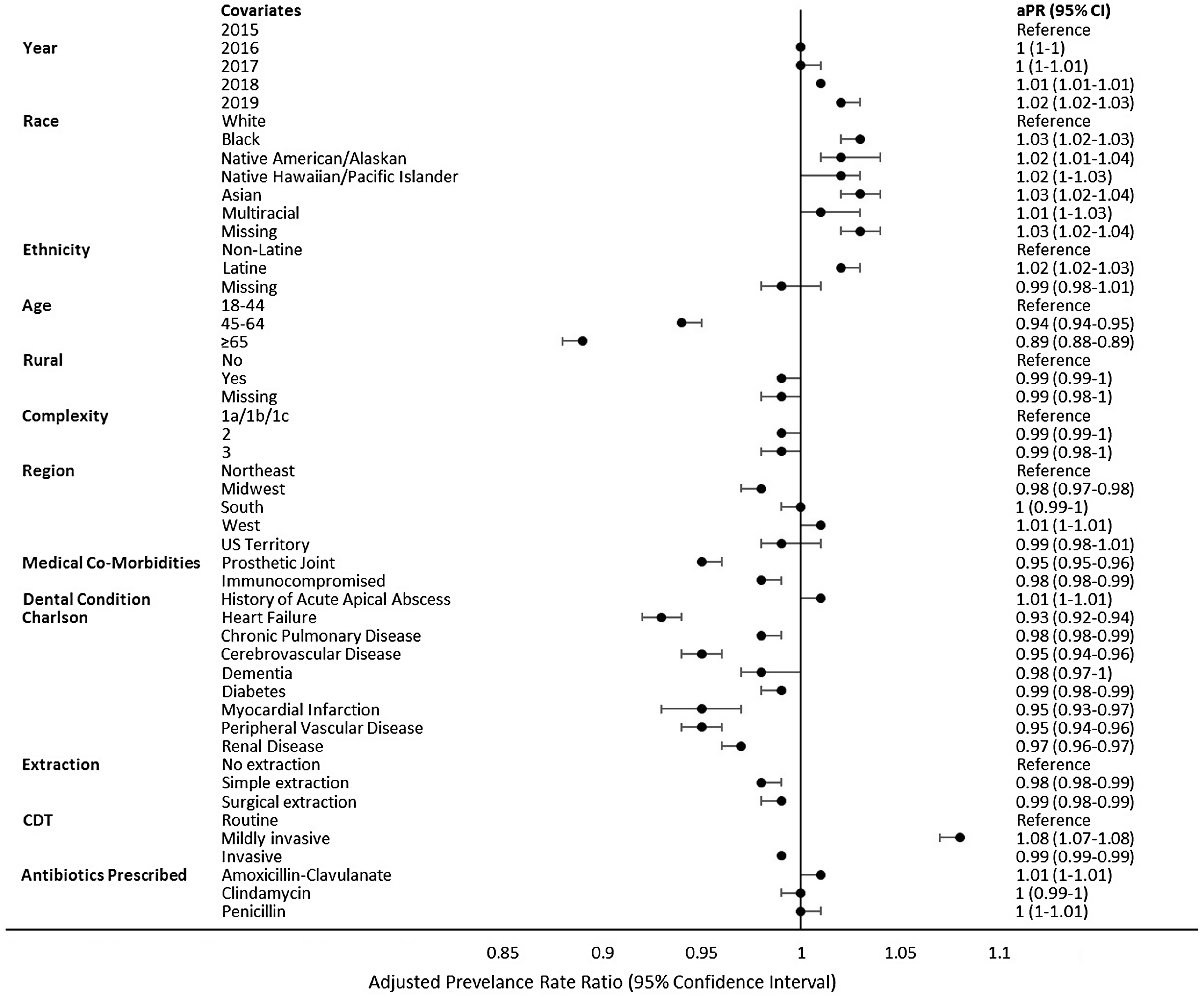
Multivariable log-linear generalized estimating equations Poisson model with robust standard errors showing the association between covariates and unnecessary antibiotic prophylaxis per the narrow criteria (cardiac condition + gingival manipulation).

**Fig. 3.**
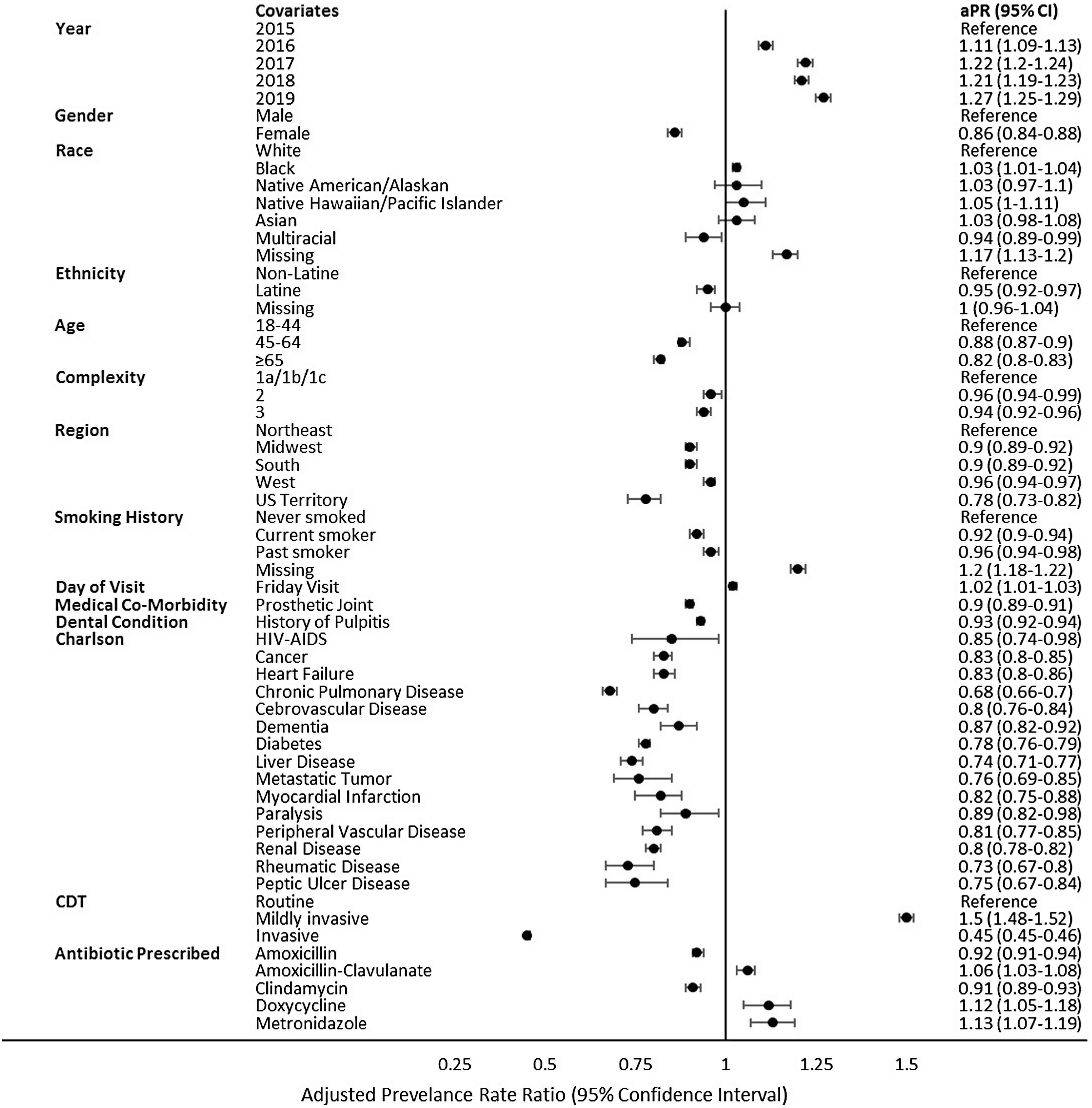
Multivariable log-linear generalized estimating equations Poisson model with robust standard errors showing the association between covariates and unnecessary antibiotic prophylaxis per the broad criteria (cardiac condition or surgical extraction and/or tooth implant or immunocompromised + gingival manipulation).

In the multivariable analysis of factors associated with unnecessary prophylaxis prescriptions, according to the narrow definition, patients in higher age categories (PR, 0.89; 95% CI, 0.88–0.89 ≥65 years; reference, 18–44 years), with prosthetic joints (PR, 0.95; 95% CI, 0.95–0.96), and with immunocompromising conditions (PR, 0.98; 95% CI, 0.98–0.99) had lower prevalence rates of unnecessary prophylaxis, whereas persons of Black (PR, 1.03; 95% CI, 1.02–1.03) race and those of Native American/Alaskan (PR, 1.02; 95% CI, 1.01–1.04), Native Hawaiian/Pacific Islander (PR, 1.02; 95% CI, 1.001–1.03), Asian (PR, 1.03; 95% CI, 1.02–1.04), multiracial (PR, 1.01; 95% CI, 1.001–1.03; reference, White), and Latine ethnicity (PR, 1.02; 95% CI, 1.02–1.03]; reference, non-Latine) had higher likelihoods of unnecessary prophylaxis (Fig. 2). Dental visits categorized as mildly invasive had a higher likelihood of unnecessary prophylaxis (PR, 1.08; 95% CI, 1.07–1.08), and invasive visits had a decreased likelihood of unnecessary prophylaxis (PR, 0.99; 95% CI, 0.99–0.99; reference, routine). Unnecessary prophylaxis prescriptions increased over time (PR, 1.02; 95% CI, 1.02–1.03; reference, 2015). Although similar predictors were identified on multivariable analysis for the broad definition (Fig. 3), differences were observed for unnecessary prophylaxis prescriptions by region (lower PR vs the Northeast region) (Supplementary Fig. 2 online).

### Sensitivity analyses

Sensitivity analyses tested the robustness of our findings. To account for missing gingival manipulation codes, only comorbidities were included in the appropriate prophylaxis definition. Removing gingival manipulation criteria increased appropriate prophylaxis slightly to 17% according to the narrow definition and 79% for the broad definition (vs 15% and 72%, respectively). Results were similar after including prosthetic joint placement within 2 years (16% was appropriate). After modifying the appropriate prophylaxis definition according to the 2003 prosthetic joint guidelines,^17^ 22% were appropriate. Stratifying the cohort by the presence or absence of a prosthetic joint, 17,143 (19%) of 92,522 patients with a prosthetic joint received an appropriate prophylaxis prescription secondary to a concomitant cardiac condition. After excluding those with a prosthetic joint, the percentage of visits with appropriate prophylaxis prescription decreased to 14% (N = 36,299 of 265,556). Compared with the primary analysis (narrow definition), similar predictors associated with unnecessary prophylaxis were identified (Supplementary Figs. 3 and 4).

## Discussion

Most antibiotics prescribed prior to a dental visit for infection prophylaxis in the VHA were likely unnecessary, according to available guidelines. These results are consistent with analyses in a commercially insured population and from other countries where 58%–81% of dental antibiotics were potentially unnecessary.^15,25–29^ Recent data indicate that antibiotic prescribing by US dentists exceeds that of dentists in Australia, Canada, and England.^30^ Although 90% of antibiotic selection was appropriate, and few broad-spectrum antibiotics were prescribed, unnecessary prescribing increased over time. Even though prophylaxis is indicated as 1 dose prior to the dental visit, only 1 in 15 antibiotics prescribed for infection prophylaxis were prescribed for 1 day (appropriate for 1 dose prior to a dental visit). In fact, the median duration was 7 days, and 1 of 4 antibiotics were prescribed for ≥10 days.

For patients with high-risk cardiac conditions, guidelines recommend antibiotics prior to invasive procedures.^7^ Meta-analyses support prophylaxis prior to extractions and implants in the prevention of postoperative complications.^9,10^ Recent evidence suggests that the risk of adverse events may not exceed the benefit in preventing complications after implant surgery.^31^ Prophylaxis prescriptions in patients with immunocompromising conditions are common, even though data supporting this practice are lacking.^11^ To estimate unnecessary prophylaxis, we applied both narrow and broad approaches to identifying indications for which antibiotic prophylaxis may be recommended in 15% and 75% of cases, respectively. This significant increase in appropriate prescribing highlights dentists’ use of antibiotics for the prevention of postsurgical local infection. Although some data support this use, no guidelines have been established. Although invasive procedures may be difficult to classify, even under the broad definition, one-quarter of patients likely received unnecessary prophylaxis. Among these patients, the major indications were for prosthetic joints (18.7%) and/or diabetes (10.8%).

Unlike other reports of national antibiotic prescribing and prescriptions by dentists,^2,32^ we observed minimal geographic variability. This finding may be due to the focus on an integrated health system (VHA) or specific indication (prophylaxis). The finding that unnecessary prophylaxis prescribing was the highest in the West, however, is discordant with previous reports of medical clinicians and population-level prescribing,^2,33^ but it is consistent with analyses of unnecessary prophylaxis prior to dental visits.^3,15^ Women have been identified to be associated with higher antibiotic prescribing rates and potentially unnecessary prophylaxis.^3,15^ However, the association of dentist prescribing by race and ethnicity has not been previously described. At the community level, Black race has been associated with higher antibiotic prescribing nationally, whereas white race has been associated with higher dental prescribing.^3,32^ In this cohort, minorities and Latines were more likely to receive unnecessary prophylaxis. These differing results may be due to a disproportionate share of comorbidities, limited healthcare access, or poor oral health.

The broad definition that accounted for guideline-recommended cardiac conditions, evidence from meta-analyses, and common practice, revealed similar factors associated with unnecessary prophylaxis. However, some differences in predictors between the adjusted models were identified. For the broad definition, Latine ethnicity was less likely and the Northeast region and dental visits occurring on Fridays were more likely to be associated with potentially unnecessary prophylaxis. Prescribing cultures, distribution of patient comorbidities, and patient and physician demands for dentists to prescribe may differ by region and patient ethnicity. Data from medical visits suggest increased unnecessary prescribing for viral respiratory infections later in the day, possibly due to decision fatigue.^34^ A similar phenomenon may occur in dentistry. Dentists may also prescribe antibiotics “just in case” complications occur, to avoid weekend calls.

Sensitivity analyses expanding our definition of appropriate antibiotics based on prior guidelines recommending prophylaxis in select patients with prosthetic joints, increased the proportion of appropriate antibiotics from 15% to 22%, which may indicate that guidelines discouraging prophylaxis prescribing in patients with prosthetic joints have not yet been adapted into practice.^18^ Pressure for dentists to prescribe antibiotics by medical clinicians may also account for the lack of guideline uptake.^35^ Other factors that may be associated with potentially unnecessary prescribing by dentists include time constraints, an aging population, dental implant placements, underinsurance, and procedural skills during emergencies.^25,35,36^ However, US-based qualitative studies identifying perceptions associated with unnecessary antibiotic prophylaxis prescriptions are limited.

This study had several limitations. It was retrospective in design and used electronic health record data, which may have included misclassified data. Collapsing dental visits into episodes of care may have inaccurately grouped some visits with an earlier unrelated visit. However, most subsequent visits were within 7 days. Furthermore, the study was limited to veterans and may not be generalizable to the US population, particularly since most veterans are male. This study only included VHA data and may have missed non-VHA encounters that could have affected the findings. However, these results significantly add to our knowledge of antibiotic prescribing because VHA dental visits include diagnostic codes, unlike the private sector, which allowed removal of antibiotics likely prescribed for infections. We were also able to identify conditions that occurred more than a decade prior to the index date, confirming an important criterion of guideline-concordance (eg, cardiac conditions).

This analysis of VHA dental data confirms previous findings with the private sector.^15^ Taken together, results from these 2 large national studies provide strong evidence that most antibiotic prophylaxis prescribed prior to dental visits is likely unnecessary, based on currently available guidelines, and that stewardship and additional guidance is needed in dentistry. The need for antibiotic stewardship has been supported by multiple policy statements, which recommend stewardship implementation in dentistry.^37–39^ Although dentists are included as part of the target audience for the CDC *Core Elements of Outpatient Antibiotic Stewardship*, there is only 1 US example of stewardship implementation in dentistry.^40^ Thus, stewards and public health officials should engage with dentists to support implementation of antibiotic stewardship. Furthermore, in this VHA study, the markedly greater proportion of prophylaxis being appropriate when a broader definition was applied indicates that use of antibiotics for patients receiving surgical extractions, dental implants, and who are immunocompromised is frequent. Thus, specific prophylaxis guidelines in these groups are urgently needed. Avoiding unnecessary antibiotics is important because prophylaxis prescribed by dentists for short durations are not without risk.^6^ Future antibiotic prophylaxis guidelines should include data on the risks of adverse events compared to the benefits of potentially decreasing postsurgical complications.

Of every 6 antibiotics prescribed for dental infection prophylaxis, 5 were inconsistent with guidelines. Unnecessary prescribing increased over time. Considering that most antibiotics prior to dental visits are for infection prophylaxis, focusing on improving appropriateness may have large implications for stewardship. Focusing on prescribing prophylaxis for prosthetic joints, prescriptions for >1 visit, and just in case antibiotics should be targeted. In addition, evidence to inform prescribing recommendations and guidelines are urgently needed to determine whether antibiotic prophylaxis prescriptions are indicated for surgical tooth extractions, dental implant placement surgery, and immunocompromising conditions, which are common reasons dentists prescribe antibiotics.

## Supplementary material

For supplementary material accompanying this paper visit https://doi.org/10.1017/ice.2021.521. click here to view supplementary material
