# Supplementary material for: Antibiotic prophylaxis prescriptions prior to dental visits in the Veterans’ Health Administration (VHA), 2015–2019
Source: Infect Control Hosp Epidemiol. 2022 Feb 22;43(11):1565–74. doi: 10.1017/ice.2021.521 (PMC9672829; doi:10.1017/ice.2021.521)
Supplement: Supplementary file 1 [file S0899823X21005213sup001.docx]

Supplemental Table 1. Oral infection diagnoses coded in dental visits excluded from the cohort.

| **Oral Infection Diagnosis** |
| --- |
| Disturbances in tooth eruption (includes impacted teeth) |
| Pulpitis |
| Necrosis of the pulp |
| Acute apical periodontitis of pulpal origin |
| Periapical abscess without sinus |
| Chronic apical periodontitis |
| Periapical abscess with sinus |
| Acute gingivitis, plaque induced |
| Periodontitis |
| Inflammatory conditions of jaw |
| Other cyst of the jaw |
| Alveolar osteitis |
| Peri-radicular pathology associated with previous endodontic treatment |
| Pulp degeneration |
| Radicular cyst |
| Other and unspecified diseases of pulp and periapical tissues |
| Dental caries extending into pulp |
| Cracked tooth |
| Cellulitis/abscess of oral tissue |
| Osteoradionecrosis of the jaw |

Supplemental Table 2. Medical diagnoses categorized as immunocompromising conditions.

| **Diagnoses included in the immunocompromising condition definition** |
| --- |
| Bone marrow, stem cell, or solid organ transplant |
| Rheumatoid arthritis |
| HIV or AIDS |
| Febrile neutropenia |
| Cancer patients undergoing immunosuppressive chemotherapy in the last 12 months |
| Inherited diseases of immunosuppression (congenital agammaglobulinemia, congenital IgA deficiency) |
| Poorly controlled diabetes with a HgbA1c >8 as the last value or as a mean of all HgbA1c over the last year |
| >85 days of systemic corticosteroid use in the prior 90 days before the index date |
| \| >1 prescription for an immunosuppressive medication dispensed within 90 days of the index date, medications were defined as Aldesleukin, Alemtuzumab, Alefacept, Azathioprine, Beclomethasone, Belatacept, Bendamustine, Betamethasone, Bevacizumab, Bexarotene, Bortezomib, Busulfan, Cabozantinib, Canakinumab, Capecitabine, Cisplatin, Cladribine, Clofarabine, Cortisone, Crizotinib, Cyclophosphamide, Cyclosporine, Dasatinib, Dexamethasone, Erlotinib, Everolimus, Fingolimod, Fludrocortisone, Flunisolide, Fluticasone, Hydrocortisone, Ibritumomab, Imatinib, Ixabepilone, Leflunomide,Lenalidomide, Mechlorethamine, Mercaptopurine, Methotrexate, Methylpredisolone, Mitoxantrone, Mometasone, Mycophenolate, Nilotinib, Obinutuzumab, Ofatumumab, Omecetazine, Pazopanib, Peginterferone alfa-2a, Pomalidomide, Ponatinib, Prednisone, Regorafenib, Rituximab, Romidepsin, Sirolimus, Sorafenib, Tacrolimus,Temozolomide, Temsirolimus, Teriflunomide, Thalidomide, Thiotepa, Tofacitainib, Triamcinolone \| \| --- \| |

| Supplemental Table 3. Unadjusted associations between demographic and medical characteristics and unnecessary antibiotic prophylaxis per the narrow criteria (cardiac condition + gingival manipulation) | | | | | | |
| --- | --- | --- | --- | --- | --- | --- |
| Variable | Value | Total | Appropriate (narrow criteria) n=53442 | Unnecessary n=304636 | Prevalence Rate Ratio (95% CI) | p-value |
| Year | 2015 | 71935 (20.1%) | 11479 (16%) | 60456 (84%) | Reference |  |
|  | 2016 | 74297 (20.7%) | 11502 (15.5%) | 62795 (84.5%) | 1.0057 (1.0012-1.0101) | 0.0124 |
|  | 2017 | 72874 (20.4%) | 10914 (15%) | 61960 (85%) | 1.0117 (1.0072-1.0161) | <0.0001 |
|  | 2018 | 70651 (19.7%) | 10017 (14.2%) | 60634 (85.8%) | 1.0212 (1.0167-1.0256) | <0.0001 |
|  | 2019 | 68321 (19.1%) | 9530 (13.9%) | 58791 (86.1%) | 1.0239 (1.0194-1.0284) | <0.0001 |
| Gender | Male | 325763 (91%) | 49722 (15.3%) | 276041 (84.7%) | Reference |  |
|  | Female | 32315 (9%) | 3720 (11.5%) | 28595 (88.5%) | 1.0443 (1.0399-1.0487) | <0.0001 |
| Race | White | 241747 (67.5%) | 38810 (16.1%) | 202937 (83.9%) | Reference |  |
|  | Black | 89475 (25%) | 11303 (12.6%) | 78172 (87.4%) | 1.0408 (1.0376-1.0439) | <0.0001 |
|  | Native American / Alaskan | 2992 (0.8%) | 423 (14.1%) | 2569 (85.9%) | 1.0228 (1.008-1.0379) | 0.0043 |
|  | Native Hawaiian / Pacific Islander | 3759 (1%) | 507 (13.5%) | 3252 (86.5%) | 1.0306 (1.0175-1.0438) | <0.0001 |
|  | Asian | 3978 (1.1%) | 422 (10.6%) | 3556 (89.4%) | 1.0649 (1.0534-1.0765) | <0.0001 |
|  | Multiracial | 4064 (1.1%) | 540 (13.3%) | 3524 (86.7%) | 1.033 (1.0205-1.0456) | <0.0001 |
|  | Missing | 12063 (3.4%) | 1437 (11.9%) | 10626 (88.1%) |  |  |
| Ethnicity | Non-Latine | 321821 (89.9%) | 48795 (15.2%) | 273026 (84.8%) | Reference |  |
|  | Latine | 29268 (8.2%) | 3688 (12.6%) | 25580 (87.4%) | 1.0302 (1.0255-1.0349) | <0.0001 |
|  | Missing | 6989 (2%) | 959 (13.7%) | 6030 (86.3%) |  |  |
| Age Categories | 18-44 | 38447 (10.7%) | 1824 (4.7%) | 36623 (95.3%) | Reference |  |
|  | 45-64 | 128429 (35.9%) | 14676 (11.4%) | 113753 (88.6%) | 0.9298 (0.9271-0.9326) | <0.0001 |
|  | >=65 | 191202 (53.4%) | 36942 (19.3%) | 154260 (80.7%) | 0.847 (0.8443-0.8496) | <0.0001 |
| Rural | No | 306122 (85.5%) | 44941 (14.7%) | 261181 (85.3%) | Reference |  |
|  | Yes | 40025 (11.2%) | 6635 (16.6%) | 33390 (83.4%) | 0.9778 (0.9733-0.9823) | <0.0001 |
|  | Missing | 11931 (3.3%) | 1866 (15.6%) | 10065 (84.4%) |  |  |
| Complexity | 1a/1b/1c | 308582 (86.2%) | 45357 (14.7%) | 263225 (85.3%) | Reference |  |
|  | 2 | 22933 (6.4%) | 3753 (16.4%) | 19180 (83.6%) | 0.9805 (0.9747-0.9863) | <0.0001 |
|  | 3 | 26563 (7.4%) | 4332 (16.3%) | 22231 (83.7%) | 0.9811 (0.9757-0.9865) | <0.0001 |
| Region | Northeast | 51817 (14.5%) | 7848 (15.1%) | 43969 (84.9%) | Reference |  |
|  | Midwest | 67002 (18.7%) | 11950 (17.8%) | 55052 (82.2%) | 0.9683 (0.9634-0.9732) | <0.0001 |
|  | South | 160298 (44.8%) | 22454 (14%) | 137844 (86%) | 1.0134 (1.0092-1.0176) | <0.0001 |
|  | West | 72967 (20.4%) | 10301 (14.1%) | 62666 (85.9%) | 1.0121 (1.0074-1.0169) | <0.0001 |
|  | U.S. Territories | 5994 (1.7%) | 889 (14.8%) | 5105 (85.2%) | 1.0037 (0.9925-1.015) | 0.5297 |
| Smoking | Never smoked | 68945 (19.3%) | 10677 (15.5%) | 58268 (84.5%) | Reference |  |
|  | Current smoker | 104679 (29.2%) | 15160 (14.5%) | 89519 (85.5%) | 1.0119 (1.0078-1.016) | <0.0001 |
|  | Past smoker | 59826 (16.7%) | 10762 (18%) | 49064 (82%) | 0.9704 (0.9656-0.9752) | <0.0001 |
|  | Missing | 124628 (34.8%) | 16843 (13.5%) | 107785 (86.5%) |  |  |
| Friday Visit | No | 296348 (82.8%) | 44555 (15%) | 251793 (85%) | Reference |  |
|  | Yes | 61730 (17.2%) | 8887 (14.4%) | 52843 (85.6%) | 1.0075 (1.0039-1.0111) | <0.0001 |
| Cardiac Condition | No | 297650 (83.1%) | 0 (0%) | 297650 (100%) | Reference |  |
|  | Yes | 60428 (16.9%) | 53442 (88.4%) | 6986 (11.6%) | 0.1156 (0.1131-0.1182) | <0.0001 |
| Prosthetic Condition | No | 265556 (74.2%) | 36299 (13.7%) | 229257 (86.3%) | Reference |  |
|  | Yes | 92522 (25.8%) | 17143 (18.5%) | 75379 (81.5%) | 0.9437 (0.9405-0.9469) | <0.0001 |
| Immunocompromised | No | 115985 (32.4%) | 11040 (9.5%) | 104945 (90.5%) | Reference |  |
|  | Yes | 242093 (67.6%) | 42402 (17.5%) | 199691 (82.5%) | 0.9116 (0.9092-0.914) | <0.0001 |
| Pulpitis | No | 157334 (43.9%) | 22005 (14%) | 135329 (86%) | Reference |  |
|  | Yes | 200744 (56.1%) | 31437 (15.7%) | 169307 (84.3%) | 0.9805 (0.9779-0.9832) | <0.0001 |
| Periodontitis | No | 329961 (92.1%) | 49077 (14.9%) | 280884 (85.1%) | Reference |  |
|  | Yes | 28117 (7.9%) | 4365 (15.5%) | 23752 (84.5%) | 0.9924 (0.9872-0.9975) | 0.0034 |
| Acute Apical Abscess | No | 346946 (96.9%) | 51934 (15%) | 295012 (85%) | Reference |  |
|  | Yes | 11132 (3.1%) | 1508 (13.5%) | 9624 (86.5%) | 1.0167 (1.0091-1.0244) | <0.0001 |
| Elixhauser Index | 0 | 121110 (33.8%) | 9436 (7.8%) | 111674 (92.2%) | Reference |  |
|  | 1 | 97805 (27.3%) | 12906 (13.2%) | 84899 (86.8%) | 0.9414 (0.9386-0.9442) | <0.0001 |
|  | 2 | 139163 (38.9%) | 31100 (22.3%) | 108063 (77.7%) | 0.8421 (0.8394-0.8449) | <0.0001 |
| Charlson Score | 0 | 267437 (74.7%) | 32429 (12.1%) | 235008 (87.9%) | Reference |  |
|  | 1 | 25775 (7.2%) | 4667 (18.1%) | 21108 (81.9%) | 0.9319 (0.9264-0.9375) | <0.0001 |
|  | 2 or more | 64866 (18.1%) | 16346 (25.2%) | 48520 (74.8%) | 0.8512 (0.8472-0.8552) | <0.0001 |
| AIDS | No | 357196 (99.8%) | 53295 (14.9%) | 303901 (85.1%) | Reference |  |
|  | Yes | 882 (0.2%) | 147 (16.7%) | 735 (83.3%) | 0.9795 (0.951-1.0088) | 0.1556 |
| Cancer | No | 339175 (94.7%) | 49653 (14.6%) | 289522 (85.4%) | Reference |  |
|  | Yes | 18903 (5.3%) | 3789 (20%) | 15114 (80%) | 0.9367 (0.9299-0.9435) | <0.0001 |
| Heart failure | No | 343050 (95.8%) | 46517 (13.6%) | 296533 (86.4%) | Reference |  |
|  | Yes | 15028 (4.2%) | 6925 (46.1%) | 8103 (53.9%) | 0.6238 (0.6146-0.6331) | <0.0001 |
| Chronic pulmonary disease | No | 332995 (93%) | 46903 (14.1%) | 286092 (85.9%) | Reference |  |
|  | Yes | 25083 (7%) | 6539 (26.1%) | 18544 (73.9%) | 0.8605 (0.8541-0.867) | <0.0001 |
| Cerebrovascular disease | No | 349350 (97.6%) | 50172 (14.4%) | 299178 (85.6%) | Reference |  |
|  | Yes | 8728 (2.4%) | 3270 (37.5%) | 5458 (62.5%) | 0.7302 (0.7184-0.7422) | <0.0001 |
| Dementia | No | 353530 (98.7%) | 52092 (14.7%) | 301438 (85.3%) | Reference |  |
|  | Yes | 4548 (1.3%) | 1350 (29.7%) | 3198 (70.3%) | 0.8247 (0.8092-0.8404) | <0.0001 |
| Diabetes | No | 310110 (86.6%) | 42055 (13.6%) | 268055 (86.4%) | Reference |  |
|  | Yes | 47968 (13.4%) | 11387 (23.7%) | 36581 (76.3%) | 0.8823 (0.8777-0.8868) | <0.0001 |
| Liver Disease | No | 348846 (97.4%) | 51474 (14.8%) | 297372 (85.2%) | Reference |  |
|  | Yes | 9232 (2.6%) | 1968 (21.3%) | 7264 (78.7%) | 0.923 (0.9132-0.933) | <0.0001 |
| Metastatic Tumor | No | 355872 (99.4%) | 53036 (14.9%) | 302836 (85.1%) | Reference |  |
|  | Yes | 2206 (0.6%) | 406 (18.4%) | 1800 (81.6%) | 0.9589 (0.94-0.9781) | <0.0001 |
| Myocardial Infarction | No | 354630 (99%) | 51831 (14.6%) | 302799 (85.4%) | Reference |  |
|  | Yes | 3448 (1%) | 1611 (46.7%) | 1837 (53.3%) | 0.624 (0.6047-0.6438) | <0.0001 |
| Paralysis | No | 356023 (99.4%) | 52922 (14.9%) | 303101 (85.1%) | Reference |  |
|  | Yes | 2055 (0.6%) | 520 (25.3%) | 1535 (74.7%) | 0.8774 (0.8555-0.8998) | <0.0001 |
| Peripheral vascular disease | No | 348546 (97.3%) | 49915 (14.3%) | 298631 (85.7%) | Reference |  |
|  | Yes | 9532 (2.7%) | 3527 (37%) | 6005 (63%) | 0.7353 (0.724-0.7467) | <0.0001 |
| Renal Disease | No | 334444 (93.4%) | 46064 (13.8%) | 288380 (86.2%) | Reference |  |
|  | Yes | 23634 (6.6%) | 7378 (31.2%) | 16256 (68.8%) | 0.7977 (0.7908-0.8047) | <0.0001 |
| Rheumatic Disease | No | 355667 (99.3%) | 52925 (14.9%) | 302742 (85.1%) | Reference |  |
|  | Yes | 2411 (0.7%) | 517 (21.4%) | 1894 (78.6%) | 0.9229 (0.9038-0.9424) | <0.0001 |
| Ulcer | No | 356591 (99.6%) | 53045 (14.9%) | 303546 (85.1%) | Reference |  |
|  | Yes | 1487 (0.4%) | 397 (26.7%) | 1090 (73.3%) | 0.8611 (0.8351-0.888) | <0.0001 |
| Extraction | No extraction | 253724 (70.9%) | 36794 (14.5%) | 216930 (85.5%) | Reference |  |
|  | Simple extraction | 51393 (14.4%) | 8327 (16.2%) | 43066 (83.8%) | 0.9801 (0.9761-0.9842) | <0.0001 |
|  | Surgical extraction | 52961 (14.8%) | 8321 (15.7%) | 44640 (84.3%) | 0.9858 (0.9819-0.9898) | <0.0001 |
| Invasive dental visit | Routine | 125091 (34.9%) | 19497 (15.6%) | 105594 (84.4%) | Reference |  |
|  | Mildly invasive | 40824 (11.4%) | 5657 (13.9%) | 35167 (86.1%) | 1.0205 (1.0158-1.0252) | <0.0001 |
|  | Invasive | 192163 (53.7%) | 28288 (14.7%) | 163875 (85.3%) | 1.0103 (1.0072-1.0133) | <0.0001 |
| Amoxicillin | No | 90252 (25.2%) | 13440 (14.9%) | 76812 (85.1%) | Reference |  |
|  | Yes | 267826 (74.8%) | 40002 (14.9%) | 227824 (85.1%) | 0.9995 (0.9963-1.0026) | 0.75 |
| Amoxicillin/Clavulanic acid | No | 339809 (94.9%) | 51264 (15.1%) | 288545 (84.9%) | Reference |  |
|  | Yes | 18269 (5.1%) | 2178 (11.9%) | 16091 (88.1%) | 1.0373 (1.0316-1.043) | <0.0001 |
| Clindamycin | No | 300079 (83.8%) | 43861 (14.6%) | 256218 (85.4%) | Reference |  |
|  | Yes | 57999 (16.2%) | 9581 (16.5%) | 48418 (83.5%) | 0.9777 (0.9739-0.9815) | <0.0001 |
| Cephalexin | No | 355190 (99.2%) | 53039 (14.9%) | 302151 (85.1%) | Reference |  |
|  | Yes | 2888 (0.8%) | 403 (14%) | 2485 (86%) | 1.0115 (0.9967-1.0265) | 0.1493 |
| Azithromycin | No | 354141 (98.9%) | 52917 (14.9%) | 301224 (85.1%) | Reference |  |
|  | Yes | 3937 (1.1%) | 525 (13.3%) | 3412 (86.7%) | 1.0189 (1.0064-1.0315) | 0.0046 |
| Penicillin | No | 334881 (93.5%) | 50602 (15.1%) | 284279 (84.9%) | Reference |  |
|  | Yes | 23197 (6.5%) | 2840 (12.2%) | 20357 (87.8%) | 1.0338 (1.0286-1.039) | <0.0001 |
| Doxycycline | No | 355584 (99.3%) | 53129 (14.9%) | 302455 (85.1%) | Reference |  |
|  | Yes | 2494 (0.7%) | 313 (12.6%) | 2181 (87.4%) | 1.0281 (1.0129-1.0436) | 0.0007 |
| Metronidazole | No | 355693 (99.3%) | 53173 (14.9%) | 302520 (85.1%) | Reference |  |
|  | Yes | 2385 (0.7%) | 269 (11.3%) | 2116 (88.7%) | 1.0432 (1.0283-1.0583) | <0.0001 |
| Fluoroquinolone | No | 357677 (99.9%) | 53392 (14.9%) | 304285 (85.1%) | Reference |  |
|  | Yes | 401 (0.1%) | 50 (12.5%) | 351 (87.5%) | 1.0289 (0.9916-1.0676) | 0.1827 |
| Other Antibiotic | No | 357549 (99.9%) | 53371 (14.9%) | 304178 (85.1%) | Reference |  |
|  | Yes | 529 (0.1%) | 71 (13.4%) | 458 (86.6%) | 1.0177 (0.9841-1.0524) | 0.3598 |

| Supplemental Table 4. Unadjusted associations between demographic and medical characteristics and unnecessary antibiotic prophylaxis per the broad criteria (cardiac condition or surgical extraction/tooth implant or immunocompromised + gingival manipulation) | | | | | | |
| --- | --- | --- | --- | --- | --- | --- |
| Variable | Value | Total | Appropriate (broad criteria) n=256591 | Inappropriate n=101487 | Prevalence Rate Ratio (95% CI) | p-value |
| Year | 2015 | 71935 (20.1%) | 53752 (74.7%) | 18183 (25.3%) | Reference | |
|  | 2016 | 74297 (20.7%) | 54067 (72.8%) | 20230 (27.2%) | 1.0772 (1.0588-1.0959) | <0.0001 |
|  | 2017 | 72874 (20.4%) | 51041 (70%) | 21833 (30%) | 1.1853 (1.1656-1.2053) | <0.0001 |
|  | 2018 | 70651 (19.7%) | 49577 (70.2%) | 21074 (29.8%) | 1.1801 (1.1603-1.2002) | <0.0001 |
|  | 2019 | 68321 (19.1%) | 48154 (70.5%) | 20167 (29.5%) | 1.1678 (1.148-1.1879) | <0.0001 |
| Gender | Male | 325763 (91%) | 233164 (71.6%) | 92599 (28.4%) | Reference | |
|  | Female | 32315 (9%) | 23427 (72.5%) | 8888 (27.5%) | 0.9676 (0.9498-0.9857) | 0.0005 |
| Race | White | 241747 (67.5%) | 173994 (72%) | 67753 (28%) | Reference | |
|  | Black | 89475 (25%) | 64039 (71.6%) | 25436 (28.4%) | 1.0143 (1.002-1.0268) | 0.0226 |
|  | Native American / Alaskan | 2992 (0.8%) | 2125 (71%) | 867 (29%) | 1.0339 (0.9772-1.094) | 0.2515 |
|  | Native Hawaiian / Pacific Islander | 3759 (1%) | 2687 (71.5%) | 1072 (28.5%) | 1.0175 (0.9669-1.0708) | 0.5101 |
|  | Asian | 3978 (1.1%) | 2762 (69.4%) | 1216 (30.6%) | 1.0907 (1.0403-1.1435) | 0.0005 |
|  | Multiracial | 4064 (1.1%) | 2983 (73.4%) | 1081 (26.6%) | 0.9491 (0.9015-0.9992) | 0.0446 |
|  | Missing | 12063 (3.4%) | 8001 (66.3%) | 4062 (33.7%) |  |  |
| Ethnicity | Non-Latine | 321821 (89.9%) | 230000 (71.5%) | 91821 (28.5%) | Reference |  |
|  | Latine | 29268 (8.2%) | 21837 (74.6%) | 7431 (25.4%) | 0.8899 (0.8719-0.9082) | <0.0001 |
|  | Missing | 6989 (2%) | 4754 (68%) | 2235 (32%) |  |  |
| Age Categories | 18-44 | 38447 (10.7%) | 25219 (65.6%) | 13228 (34.4%) | Reference | |
|  | 45-64 | 128429 (35.9%) | 92231 (71.8%) | 36198 (28.2%) | 0.8192 (0.8059-0.8327) | <0.0001 |
|  | >=65 | 191202 (53.4%) | 139141 (72.8%) | 52061 (27.2%) | 0.7914 (0.7791-0.8038) | <0.0001 |
| Rural | No | 306122 (85.5%) | 218950 (71.5%) | 87172 (28.5%) | Reference | |
|  | Yes | 40025 (11.2%) | 28573 (71.4%) | 11452 (28.6%) | 1.0048 (0.9884-1.0214) | 0.5719 |
|  | Missing | 11931 (3.3%) | 9068 (76%) | 2863 (24%) | |  |
| Complexity | 1a/1b/1c | 308582 (86.2%) | 222542 (72.1%) | 86040 (27.9%) | Reference | |
|  | 2 | 22933 (6.4%) | 16115 (70.3%) | 6818 (29.7%) | 1.0663 (1.0444-1.0886) | <0.0001 |
|  | 3 | 26563 (7.4%) | 17934 (67.5%) | 8629 (32.5%) | 1.1651 (1.144-1.1865) | <0.0001 |
| Region | Northeast | 51817 (14.5%) | 36845 (71.1%) | 14972 (28.9%) | Reference | |
|  | Midwest | 67002 (18.7%) | 48882 (73%) | 18120 (27%) | 0.936 (0.9189-0.9533) | <0.0001 |
|  | South | 160298 (44.8%) | 114718 (71.6%) | 45580 (28.4%) | 0.9841 (0.9689-0.9996) | 0.0446 |
|  | West | 72967 (20.4%) | 51386 (70.4%) | 21581 (29.6%) | 1.0236 (1.0058-1.0417) | 0.0091 |
|  | U.S. Territories | 5994 (1.7%) | 4760 (79.4%) | 1234 (20.6%) | 0.7125 (0.6767-0.7502) | <0.0001 |
| Smoking | Never smoked | 68945 (19.3%) | 49964 (72.5%) | 18981 (27.5%) | Reference | |
|  | Current smoker | 104679 (29.2%) | 79293 (75.7%) | 25386 (24.3%) | 0.8809 (0.8668-0.8952) | <0.0001 |
|  | Past smoker | 59826 (16.7%) | 44724 (74.8%) | 15102 (25.2%) | 0.9169 (0.9002-0.9339) | <0.0001 |
|  | Missing | 124628 (34.8%) | 82610 (66.3%) | 42018 (33.7%) | |  |
| Friday Visit | No | 296348 (82.8%) | 213201 (71.9%) | 83147 (28.1%) | Reference | |
|  | Yes | 61730 (17.2%) | 43390 (70.3%) | 18340 (29.7%) | 1.0589 (1.0448-1.0732) | <0.0001 |
| Cardiac Condition | No | 297650 (83.1%) | 203149 (68.3%) | 94501 (31.7%) | Reference | |
|  | Yes | 60428 (16.9%) | 53442 (88.4%) | 6986 (11.6%) | 0.3641 (0.356-0.3725) | <0.0001 |
| Prosthetic Condition | No | 265556 (74.2%) | 189773 (71.5%) | 75783 (28.5%) | Reference | |
|  | Yes | 92522 (25.8%) | 66818 (72.2%) | 25704 (27.8%) | 0.9735 (0.9619-0.9853) | <0.0001 |
| Immunocompromised | No | 115985 (32.4%) | 38679 (33.3%) | 77306 (66.7%) | Reference | |
|  | Yes | 242093 (67.6%) | 217912 (90%) | 24181 (10%) | 0.1499 (0.148-0.1518) | <0.0001 |
| Pulpitis | No | 157334 (43.9%) | 110354 (70.1%) | 46980 (29.9%) | Reference | |
|  | Yes | 200744 (56.1%) | 146237 (72.8%) | 54507 (27.2%) | 0.9093 (0.8999-0.9189) | <0.0001 |
| Periodontitis | No | 329961 (92.1%) | 235976 (71.5%) | 93985 (28.5%) | Reference | |
|  | Yes | 28117 (7.9%) | 20615 (73.3%) | 7502 (26.7%) | 0.9367 (0.9181-0.9558) | <0.0001 |
| Acute Apical Abscess | No | 346946 (96.9%) | 248679 (71.7%) | 98267 (28.3%) | Reference | |
|  | Yes | 11132 (3.1%) | 7912 (71.1%) | 3220 (28.9%) | 1.0213 (0.9915-1.0519) | 0.1649 |
| Elixhauser Index | 0 | 121110 (33.8%) | 69682 (57.5%) | 51428 (42.5%) | Reference | |
|  | 1 | 97805 (27.3%) | 71398 (73%) | 26407 (27%) | 0.6358 (0.6281-0.6436) | <0.0001 |
|  | 2 | 139163 (38.9%) | 115511 (83%) | 23652 (17%) | 0.4002 (0.3949-0.4056) | <0.0001 |
| Charlson Score | 0 | 267437 (74.7%) | 183725 (68.7%) | 83712 (31.3%) | Reference | |
|  | 1 | 25775 (7.2%) | 19903 (77.2%) | 5872 (22.8%) | 0.7278 (0.7111-0.7449) | <0.0001 |
|  | 2 or more | 64866 (18.1%) | 52963 (81.6%) | 11903 (18.4%) | 0.5862 (0.5763-0.5964) | <0.0001 |
| HIV/AIDS | No | 357196 (99.8%) | 255881 (71.6%) | 101315 (28.4%) | Reference | |
|  | Yes | 882 (0.2%) | 710 (80.5%) | 172 (19.5%) | 0.6875 (0.6012-0.7863) | <0.0001 |
| Cancer | No | 339175 (94.7%) | 241305 (71.1%) | 97870 (28.9%) | Reference | |
|  | Yes | 18903 (5.3%) | 15286 (80.9%) | 3617 (19.1%) | 0.6631 (0.6437-0.6832) | <0.0001 |
| Heart failure | No | 343050 (95.8%) | 243859 (71.1%) | 99191 (28.9%) | Reference | |
|  | Yes | 15028 (4.2%) | 12732 (84.7%) | 2296 (15.3%) | 0.5284 (0.5087-0.5489) | <0.0001 |
| Chronic pulmonary disease | No | 332995 (93%) | 235352 (70.7%) | 97643 (29.3%) | Reference | |
|  | Yes | 25083 (7%) | 21239 (84.7%) | 3844 (15.3%) | 0.5226 (0.5074-0.5383) | <0.0001 |
| Cerebrovascular disease | No | 349350 (97.6%) | 249290 (71.4%) | 100060 (28.6%) | Reference | |
|  | Yes | 8728 (2.4%) | 7301 (83.7%) | 1427 (16.3%) | 0.5708 (0.5442-0.5987) | <0.0001 |
| Dementia | No | 353530 (98.7%) | 252850 (71.5%) | 100680 (28.5%) | Reference | |
|  | Yes | 4548 (1.3%) | 3741 (82.3%) | 807 (17.7%) | 0.6231 (0.5851-0.6634) | <0.0001 |
| Diabetes | No | 310110 (86.6%) | 217844 (70.2%) | 92266 (29.8%) | Reference | |
|  | Yes | 47968 (13.4%) | 38747 (80.8%) | 9221 (19.2%) | 0.6461 (0.6339-0.6586) | <0.0001 |
| Liver Disease | No | 348846 (97.4%) | 248795 (71.3%) | 100051 (28.7%) | Reference | |
|  | Yes | 9232 (2.6%) | 7796 (84.4%) | 1436 (15.6%) | 0.5423 (0.517-0.5689) | <0.0001 |
| Metastatic Tumor | No | 355872 (99.4%) | 254711 (71.6%) | 101161 (28.4%) | Reference | |
|  | Yes | 2206 (0.6%) | 1880 (85.2%) | 326 (14.8%) | 0.5199 (0.4702-0.5747) | <0.0001 |
| Myocardial Infarction | No | 354630 (99%) | 253618 (71.5%) | 101012 (28.5%) | Reference | |
|  | Yes | 3448 (1%) | 2973 (86.2%) | 475 (13.8%) | 0.4836 (0.4448-0.5259) | <0.0001 |
| Paralysis | No | 356023 (99.4%) | 254918 (71.6%) | 101105 (28.4%) | Reference | |
|  | Yes | 2055 (0.6%) | 1673 (81.4%) | 382 (18.6%) | 0.6546 (0.5979-0.7167) | <0.0001 |
| Peripheral vascular disease | No | 348546 (97.3%) | 248565 (71.3%) | 99981 (28.7%) | Reference | |
|  | Yes | 9532 (2.7%) | 8026 (84.2%) | 1506 (15.8%) | 0.5508 (0.5257-0.5771) | <0.0001 |
| Renal Disease | No | 334444 (93.4%) | 236868 (70.8%) | 97576 (29.2%) | Reference | |
|  | Yes | 23634 (6.6%) | 19723 (83.5%) | 3911 (16.5%) | 0.5672 (0.5509-0.5839) | <0.0001 |
| Rheumatic Disease | No | 355667 (99.3%) | 254589 (71.6%) | 101078 (28.4%) | Reference | |
|  | Yes | 2411 (0.7%) | 2002 (83%) | 409 (17%) | 0.5969 (0.5464-0.6521) | <0.0001 |
| Ulcer | No | 356591 (99.6%) | 255319 (71.6%) | 101272 (28.4%) | Reference | |
|  | Yes | 1487 (0.4%) | 1272 (85.5%) | 215 (14.5%) | 0.5091 (0.4499-0.5762) | <0.0001 |
| Extraction | No extraction | 253724 (70.9%) | 166316 (65.5%) | 87408 (34.5%) | Reference | |
|  | Simple extraction | 51393 (14.4%) | 37314 (72.6%) | 14079 (27.4%) | 0.7952 (0.7833-0.8073) | <0.0001 |
|  | Surgical extraction | 52961 (14.8%) | 52961 (100%) | 0 (0%) | . (.-.) | <0.0001 |
| Invasive visit | Routine | 125091 (34.9%) | 78108 (62.4%) | 46983 (37.6%) | Reference | |
|  | Mildly invasive | 40824 (11.4%) | 19027 (46.6%) | 21797 (53.4%) | 1.4216 (1.4053-1.4381) | <0.0001 |
|  | Invasive | 192163 (53.7%) | 159456 (83%) | 32707 (17%) | 0.4532 (0.4477-0.4587) | <0.0001 |
| Amoxicillin | No | 90252 (25.2%) | 64177 (71.1%) | 26075 (28.9%) | Reference | |
|  | Yes | 267826 (74.8%) | 192414 (71.8%) | 75412 (28.2%) | 0.9746 (0.9631-0.9862) | <0.0001 |
| Amoxicillin/Clavulanic acid | No | 339809 (94.9%) | 243369 (71.6%) | 96440 (28.4%) | Reference | |
|  | Yes | 18269 (5.1%) | 13222 (72.4%) | 5047 (27.6%) | 0.9734 (0.9503-0.9971) | 0.0279 |
| Clindamycin | No | 300079 (83.8%) | 214567 (71.5%) | 85512 (28.5%) | Reference | |
|  | Yes | 57999 (16.2%) | 42024 (72.5%) | 15975 (27.5%) | 0.9666 (0.9528-0.9805) | <0.0001 |
| Cephalexin | No | 355190 (99.2%) | 254690 (71.7%) | 100500 (28.3%) | Reference | |
|  | Yes | 2888 (0.8%) | 1901 (65.8%) | 987 (34.2%) | 1.2079 (1.1479-1.2709) | <0.0001 |
| Azithromycin | No | 354141 (98.9%) | 253815 (71.7%) | 100326 (28.3%) | Reference | |
|  | Yes | 3937 (1.1%) | 2776 (70.5%) | 1161 (29.5%) | 1.0409 (0.9916-1.0928) | 0.1095 |
| Penicillin | No | 334881 (93.5%) | 240473 (71.8%) | 94408 (28.2%) | Reference | |
|  | Yes | 23197 (6.5%) | 16118 (69.5%) | 7079 (30.5%) | 1.0825 (1.0609-1.1045) | <0.0001 |
| Doxycycline | No | 355584 (99.3%) | 254940 (71.7%) | 100644 (28.3%) | Reference | |
|  | Yes | 2494 (0.7%) | 1651 (66.2%) | 843 (33.8%) | 1.1942 (1.1301-1.262) | <0.0001 |
| Metronidazole | No | 355693 (99.3%) | 255007 (71.7%) | 100686 (28.3%) | Reference | |
|  | Yes | 2385 (0.7%) | 1584 (66.4%) | 801 (33.6%) | 1.1865 (1.1211-1.2556) | <0.0001 |
| Fluoroquinolone | No | 357677 (99.9%) | 256312 (71.7%) | 101365 (28.3%) | Reference | |
|  | Yes | 401 (0.1%) | 279 (69.6%) | 122 (30.4%) | 1.0735 (0.9258-1.2449) | 0.3466 |
| Other Antibiotic | No | 357549 (99.9%) | 256236 (71.7%) | 101313 (28.3%) | Reference | |
|  | Yes | 529 (0.1%) | 355 (67.1%) | 174 (32.9%) | 1.1608 (1.0277-1.3112) | 0.0232 |

Supplemental Figure 1. Frequency in the days between dental visits for episodes of care


Supplemental Figure 2. Multivariable log-linear generalized estimating equations Poisson model with robust standard errors showing the association between covariates and unnecessary antibiotic prophylaxis per the 2003 prosthetic joint infection prophylaxis guidelines [22]


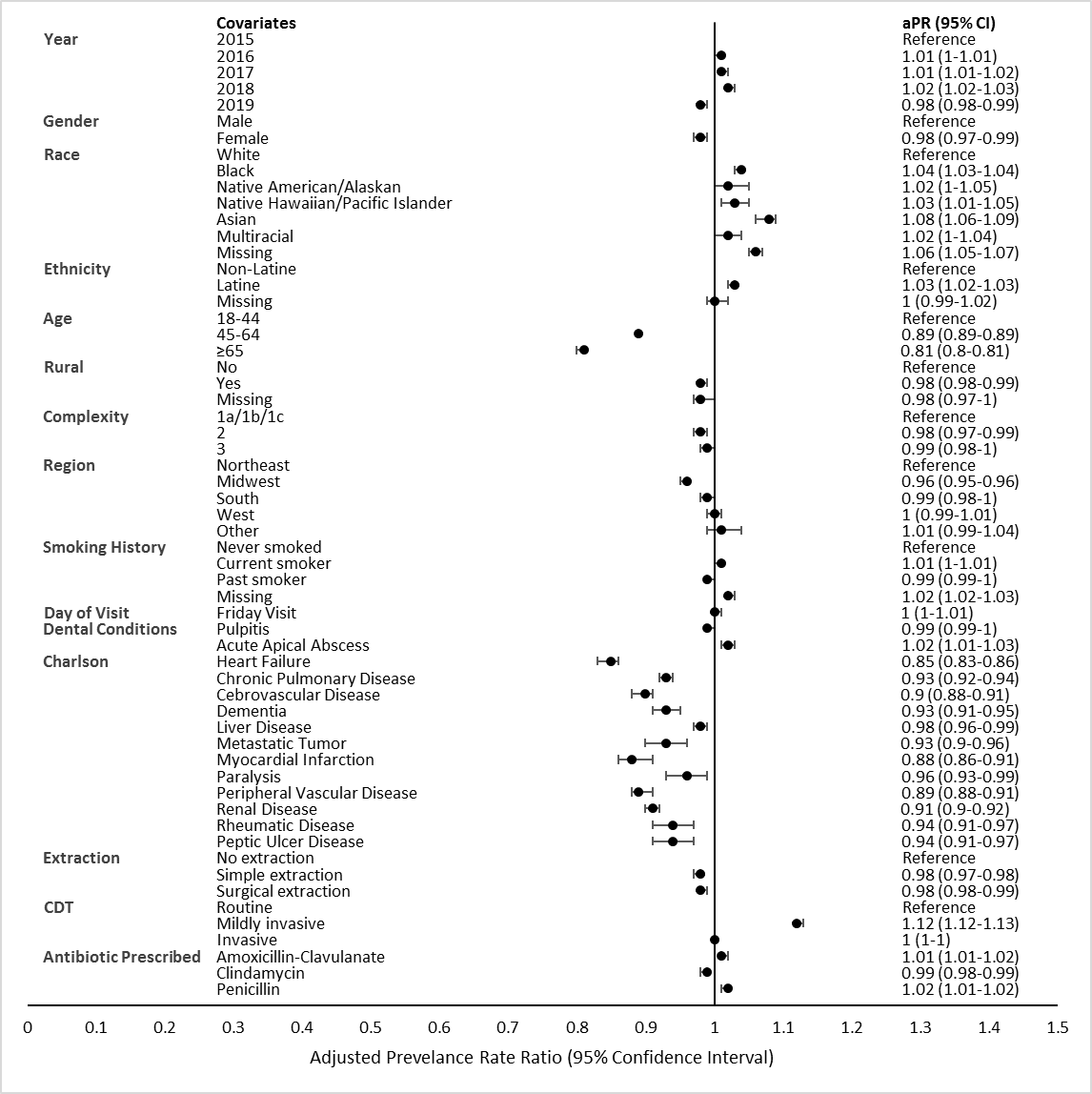


Supplemental Figure 3. Multivariable log-linear generalized estimating equations Poisson model with robust standard errors showing the association between covariates and unnecessary antibiotic prophylaxis per the narrow criteria (cardiac condition + gingival manipulation) in patients without prosthetic joints.

Supplemental Figure 4. Multivariable log-linear generalized estimating equations Poisson model with robust standard errors showing the association between covariates and unnecessary antibiotic prophylaxis per the narrow criteria (cardiac condition + gingival manipulation) in patients with prosthetic joints.
